# Covid-19-Associated Pulmonary Aspergillosis: The Other Side of the Coin

**DOI:** 10.3390/vaccines8040713

**Published:** 2020-12-01

**Authors:** Claudio Costantini, Frank L. van de Veerdonk, Luigina Romani

**Affiliations:** 1 Department of Experimental Medicine, University of Perugia, 06132 Perugia, Italy; claudio.costantini@unipg.it; 2 Department of Internal Medicine, Radboud University Medical Center, 6525 GA Nijmegen, The Netherlands; frank.vandeveerdonk@radboudumc.nl

**Keywords:** COVID-19, *Aspergillus*, anakinra, Aryl Hydrocarbon Receptor, thymosin alpha 1

## Abstract

The immune response to severe acute respiratory syndrome coronavirus 2 (SARS-CoV-2) is a critical factor in the clinical presentation of COVID-19, which may range from asymptomatic to a fatal, multi-organ disease. A dysregulated immune response not only compromises the ability of the host to resolve the viral infection, but may also predispose the individual to secondary bacterial and fungal infections, a risk to which the current therapeutic immunomodulatory approaches significantly contribute. Among the secondary infections that may occur in COVID-19 patients, coronavirus-associated pulmonary aspergillosis (CAPA) is emerging as a potential cause of morbidity and mortality, although many aspects of the disease still remain unresolved. With this opinion, we present the current view of CAPA and discuss how the same mechanisms that underlie the dysregulated immune response in COVID-19 increase susceptibility to *Aspergillus* infection. Likewise, resorting to endogenous pathways of immunomodulation may not only restore immune homeostasis in COVID-19 patients, but also reduce the risk for aspergillosis. Therefore, CAPA represents the other side of the coin in COVID-19 and our advances in the understanding and treatment of the immune response in COVID-19 should represent the framework for the study of CAPA.

## 1. Introduction

The coronavirus disease 2019 (COVID-19) is caused by the severe acute respiratory syndrome coronavirus 2 (SARS-CoV-2), which interacts with heparan sulfate and angiotensin-converting enzyme 2 (ACE2) [1] to penetrate susceptible tissues. The nose shows the highest expression of ACE2 along the respiratory tract [2] and represents a permissive entry site to the virus that subsequently translocates to the lungs, likely by aspiration, to ignite pathogenesis [2]. However, viral tropism is not restricted to the respiratory tract and the virus can be detected in multiple organs, such as the liver, brain, and kidneys [3]. The clinical presentation of COVID-19 is variable, ranging from asymptomatic to a fatal, multi-organ disease, and multiple risk factors determine an individual’s prognosis. Such risk factors include both demographic characteristics, i.e., age ([4] and refs therein) and gender [5], and clinical aspects, i.e., the presence of co-morbidities, such as diabetes [6], chronic obstructive pulmonary disease [7], cardiovascular disease [8], and cancer [9]. An increasing risk for COVID-19 patients is represented by the occurrence of co-infections and superinfections, which may deteriorate the clinical picture [10,11]. Indeed, bacterial, fungal, and viral infections have been detected in COVID-19 patients, although at a low incidence [12]. Among the superinfections that may occur in COVID-19 patients, fungal pathogens are increasingly being recognized as an emergent threat [13], although many aspects still remain unclear from a diagnostic, clinical, therapeutic, and mechanistic point of view.

With this, we will present current evidence of invasive fungal infections in COVID-19 patients and existing debate on clinical features of the disease. We will then provide possible mechanistic explanations, linking the dysregulation of the immune response in COVID-19 patients with the increased susceptibility to infection. Finally, we will discuss potential therapeutic approaches that by re-establishing a homeostatic response of the immune system, would reduce the risk of invasive fungal infections.

## 2. Coronavirus-Associated Pulmonary Aspergillosis (CAPA)

Since the first indications of the presence of *Aspergillus* in specimens from COVID-19 patients in the early descriptions of Chinese patients [14,15,16,17], reports on coronavirus-associated pulmonary aspergillosis (CAPA) have appeared in the literature [18,19,20,21,22] and increased steadily, such that more than 100 cases have now been described worldwide in intensive care units (ICU) treating COVID-19 patients [13]. However, defining the incidence of CAPA remains an open question, which is hampered by diagnostic difficulties, which are widely discussed in the literature, such as in [13,23,24]. These issues include the general absence of host factors, as defined by the European Organization for Research and Treatment of Cancer and the Mycoses Study Group [25], in COVID-19 patients, the challenges in performing bronchoscopy and autopsy for the risk of generating potentially infective aerosol, and the low sensitivity of galactomannan testing in non-neutropenic patients. Alternative methods, such as endotracheal aspirates, may provide confounding results for the inability to distinguish between colonization and invasive aspergillosis. Similarly, β-D-glucan testing is not specific for *Aspergillus* infection and may require further confirmation. Recent extensive diagnosic testing has been performed in 719 critically ill COVID-19 patients. In a subset of 61 patients for whom both serum and respiratory samples were available, rates of 5% (3/61) and 15% (10/61) of proven/probable and possible CAPA, respectively, were reported [26].

Notwithstanding these complications, the presence of *Aspergillus* in COVID-19 patients has parallels with the aspergillosis observed in patients admitted to the ICU with severe influenza or influenza-associated pulmonary aspergillosis (IAPA) [27]. Indeed, a study comparing IAPA and CAPA identified a similar incidence of invasive aspergillosis in influenza and COVID-19 patients, the overall absence of host risk factors, a similar timing of diagnosis, and a poor prognosis [28]. Accordingly, it has been proposed that the same IAPA definitions may apply to CAPA [27], although a more specific definition has recently been suggested [29]. In any case, the pathogenesis of IAPA and CAPA seems to differ for the distinct viral ability to induce tissue damage and immunomodulatory effects [27]. For instance, influenza and SARS-CoV-2 viruses bind to different receptors with distinct distribution along the respiratory tract. The human influenza virus binds sialic acids attached to galactose by an alpha(2,6) linkage commonly also present in large airways as opposed to SARS-CoV-2 receptors, which may correlate with an increased risk of invasive *Aspergillus* tracheobronchitis in IAPA compared to CAPA [27]. Second, influenza virus has a direct effect of antifungal host defence mechanisms by inhibiting the NADPH oxidase, while to date, such activities have not been described for SARS-CoV-2 [27]. These differences make CAPA a distinct clinical entity, at least in the etiology of aspergillosis, and it is debated whether COVID-19 actually represents a risk factor for aspergillosis. Indeed, a major role may be played by the therapy of COVID-19. For instance, the use of tocilizumab, a monoclonal antibody against the interleukin-6 receptor, may prevent Th17 responses and favor aspergillosis. Similarly, chronic steroid treatment may impair host defenses, including LC3-associated phagocytosis [30]. Additional risk factors, such as lung damage [31] or structural defects [22] or broad-spectrum antibiotics usage [32], may play a role in the development of aspergillosis. However, it cannot be excluded that SARS-CoV-2 infection and the dysregulated immune response may favor the creation of conditions permissive for the growth of *Aspergillus* [27].

All in all, available evidence suggest that CAPA, among the potential secondary infections, may represent a cause of mortality and morbidity in critically ill COVID-19 patients and identifying risk factors that depend on both the disease and treatment is crucial for therapeutic purposes.

## 3. Immune Response in COVID-19

Following penetration in susceptible tissues, SARS-CoV-2 elicits an immune response that may develop along different trajectories, leading to a wide spectrum of clinical presentations. In mild COVID-19, there is a strong activation of the innate immune compartment, with a prominent role for HLA-DR^hi^CD11c^hi^ inflammatory monocytes, with high expression of interferon (IFN)-stimulated genes, while severe patients are marked by the presence of dysfunctional monocytes and neutrophils [33]. The reduced antiviral type I and III IFNs response is opposed by the abundant production of inflammatory cytokines [34] and a cytokine storm may contribute to the deterioration of severe COVID-19 patients [35,36,37]. The defective IFN response may result from multiple causes, including the presence of autoantibodies [38] or inborn errors [39,40]. The adaptive immune system is also compromised in severe patients with insurgence of lymphopenia, caused by unbalanced differentiation from hematopoietic precursors in the presence of emergency myelopoiesis, impaired recruitment and activation, along with the presence of an exhausted phenotype [41]. In addition, lymphopenia may impair immunological memory [41]. In contrast, patients with asymptomatic or mild COVID-19 develop a strong T cell immunity [42]. Humoral immunity has also been linked with the outcome of COVID-19 infection. Indeed, moderate and severe COVID-19 patients develop IgG and IgM responses, but survivors and non-survivors differ in their development of humoral responses, with the latter showing defective development and impaired IgG responses [43].

The advances in the definition of the immune response in COVID-19 patients allows one to discuss whether the dysregulation observed in severe patients may result in permissive conditions for the development of secondary infections, such as invasive fungal infections. The discussion on the host antifungal mechanisms potentially subverted by the dysregulated immune response can be taken at multiple levels, including soluble mediators and immune cells. A defective type I IFN response may impair the protection against *Aspergillus*, as supported by multiple lines of evidence. For instance, the expression of type III IFNs, primed by type I IFNs, activate the antifungal response of neutrophils [44] and mice with chronic granulomatous disease (CGD), characterized by impaired phagocytic oxidase activity, and who are susceptible to invasive aspergillosis, are protected by stimulation of type I IFN [45]. In addition, plasmacytoid DCs, known to produce high amounts of type I IFNs upon viral infection, participate in the defense against *A. fumigatus* and IFN-α/βR^−/−^ mice were more susceptible to invasive aspergillosis than wild-type mice [46]. Similarly, an overproduction of inflammatory cytokines may damage the lung and promote aspergillosis [47,48,49]. With regards to immune cells, the neutrophils observed in severe patients are characterized by impaired oxidative burst [33], which may reflect in a reduced ability to protect from *Aspergillus*, similar to the condition observed in CGD. In addition, an excessive Th17 response in COVID-19 patients [50,51] may change the protective antifungal role of Th17 cells into a detrimental role [52].

In conclusion, severe COVID-19 is associated with a dysregulated immune response that may not only impact the clinical deterioration of patients, but also modulate the susceptibility to secondary infections, for instance by impairing host antifungal defences and increasing the risk of *Aspergillus* infection.

## 4. Restoring Immune Homeostasis in COVID-19 to Prevent CAPA

Current therapeutic strategies to limit the pathology associated with an increased inflammatory response are associated with side effects, including an impaired ability to respond to concomitant infections that are consequent to immune suppression. For instance, tocilizumab is associated with a higher prevalence of infection [53] and the same applies to chronic steroid treatment. An alternative strategy would be to re-equilibrate the immune response by resorting to endogenous pathways of immunomodulation ([54] and Di Stadio et al., submitted). For instance, one of the features of COVID-19 is represented by the production of the inflammatory cytokine IL-1. Epithelial damage causes the release of IL-1α, which induces the recruitment of neutrophils and monocytes and the production of IL-1β [55]. In addition, innate sensing will result in additional production of IL-1β by the NLRP3 inflammasome, creating an amplification loop and a cytokine cascade, with production of IL-6, which may turn detrimental [55]. Targeting the NLRP3/IL-1 pathway by administering anakinra, the recombinant version of IL-1 receptor antagonist might block this noxious circuit and has proved beneficial in COVID-19 patients with a favorable safety profile [54,56,57,58,59]. It is arguable that while restoring a homeostatic immune response in severe COVID-19 patients, anakinra could also reduce susceptibility to secondary infections, including aspergillosis. In this regard, we have previously shown that anakinra protected against aspergillosis in cystic fibrosis [60] and CGD [61], both characterized by unbalanced inflammasome activation and susceptibility to *Aspergillus* infection. As another example, the activation of the aryl hydrocarbon receptor (AhR), a xenobiotic receptor, was involved in the modulation of the immune response [62]. AhR activation has been linked to mucosal protection by stimulating the production of IL-22 and potentiating the barrier functons in the gut [63]. A similar activity in the respiratory tract would be beneficial to protect from mucosal damage and re-establish protection against infection [64]. Although a recent report has associated AhR activation with lung pathogenesis in COVID-19 [65], the multiplicity of ligands and the different effects upon AhR binding do not allow definitive conclusions on the role of AhR and further studies will be required to determine whether activation is protective against COVID-19 and potential secondary infections, such as with *Aspergillus*.

Another example is represented by thymosin α1, an endogenous thymic peptide with a wide range of immunomodulatory activities [66] and the capacity to balance a dysregulated immune response in a context-dependent manner. Indeed, thymosin α1 could either be immunostimulatory, such as in cancer and immune deficiency, or promote tolerance in inflammatory conditions, for instance by inducing the indoleamine 2,3-dioxygenase 1 pathway [67,68,69] or promoting autophagy [68]. The latter process is increasingly being recognized as a regulator of lung health and protection against microbial infection, with potential relevance for a variety of lung diseases, including COVID-19 [70]. Of interest, thymosin α1 has already proven to be beneficial in the protection against viral [71] and fungal [72] infections, by promoting an IFN response and a protective Th1 resistance, respectively. Its potential efficacy in critical and severe COVID-19 patients, along with an excellent safety profile, has been assessed [54,73,74] and a reversal of lymphopenia and T cell exhaustion suggested a therapeutic effect. It appears that normalization of the adaptive immune response may also be protective against secondary infections, such as aspergillosis [72]. Interestingly, thymosin α1 was not protective against COVID-19 when used in prophylaxis [75], suggesting that thymosin α1 directly works on the suppressed adaptive immune system once the disease has developed. However, thymosin α1 could still be effective in non-exposed individuals if used in combination with a vaccine [76]. The development of a vaccine is recognized as a priority to halt the pandemic and a huge effort is devoted to this purpose [77]. It is known that thymosin α1 is able to enhance the immune response to vaccination, including specific immune suppressed populations, such as elderly people [76], thus representing a potential adjuvant in the use of a SARS-CoV-2 vaccine. In addition, vaccines are also expected to reduce the COVID-19-associated complications, including secondary infections. Therefore, thymosin α1 represents an endogenous immunomodulatory molecule with multiple applications in COVID-19, ranging from its ability to restore immune homeostasis in critical and severe COVID-19 patients to the boosting of the immune response to vaccination before infection.

In summary, selective targeting of endogenous immune pathways dysregulated in COVID-19 patients may prove beneficial not only to revert the alterations of the immune response in severe cases, but also to reduce the susceptibility to superinfection, including CAPA. However, the timing of this intervention is crucial since it should be initiated in the early stages of COVID-19 with clear immune dysregulation. A serum cytokine profile along with the measurements of selected metabolites and clinical parameters would be instrumental to detect early derailment of the immune response and indicate a proper window of intervention [78].

## 5. Conclusions

COVID-19 still represents an important threat for human health. A huge effort has helped to tackle the variable presentation of the disease from a mechanistic perspective and the differential engagement of the immune system emerges as a key factor underlying this complexity. At the same time, a dysregulated immune system and the available treatments may open the door for additional threats and the increased susceptibility to secondary infections is increasingly being recognized. Among the superinfections, CAPA plays a critical role and the dissection of mechanistic events resulting in increased susceptibility to *Aspergillus* infection are just beginning to be unraveled. The possibility to resort to endogenous pathways of immune regulation, as discussed in this paper, may provide the ability to restore the immune alterations resulting from SARS-CoV-2 invasion with protection against subsequent infection, thus opening up novel opportunities for intervention.
